# Supplementary material for: Aquatic sloths (Thalassocnus) from the Miocene of Chile and the evolution of marine mammal herbivory in the Pacific Ocean
Source: PeerJ. 2025 Oct 2;13:e19897. doi: 10.7717/peerj.19897 (PMC12497401; doi:10.7717/peerj.19897)
Supplement: Supplemental Information 1 — Measurements (in mm) to compare the degree of development of the pronator ridge in the radius of Thalassocnus spp. (modified from Amson et al., 2015a:table 16). [file peerj-13-19897-s001.docx]

| **TABLE S1.** Measurements (in mm) to compare the degree of development of the pronator ridge in the radius of *Thalassocnus* spp. (modified from Amson et al., 2015a:table 16). | | | | | |
| --- | --- | --- | --- | --- | --- |
| **Taxon** | **Specimen no.** | **Depth at midshaft (D)** | **Total length (L)** | **D/L** | **Source** |
| *T. antiquus* | MUSM 228 | 34.1 | 267 | 0.13 | Amson et al., 2015a |
| *T. natans* | MNHN.F.SAS734 | 37 | 284.8 | 0.13 | Amson et al., 2015a |
|  | MUSM 2059 | 34.9 | 264 | 0.13 | Amson et al., 2015a |
|  | MPC 704-A | 37 | 264 | 0.14 | This work |
| *T. littoralis* | MNHN.F.SAS56 | 37.7 | 268.5 | 0.14 | Amson et al., 2015a |
|  | MNHN.F.SAS801 | 41 | 261.8 | 0.16 | Amson et al., 2015a |
|  | MNHN.F.SAS802 | 33.9 | 259 | 0.13 | Amson et al., 2015a |
|  | MUSM 223 | 42 | 292.5 | 0.14 | Amson et al., 2015a |
|  | MUSM 443 | 42.9 | 250.2 | 0.17 | Amson et al., 2015a |
| *T. carolomartini* | MUSM 1995 | 49.5 | 302 | 0.16 | Amson et al., 2015a |
| *T. yaucensis* | MUSM 37 | 49.4 | 250.5 | 0.20 | Amson et al., 2015a |
