# Supplementary material for: Aquatic sloths (Thalassocnus) from the Miocene of Chile and the evolution of marine mammal herbivory in the Pacific Ocean
Source: PeerJ. 2025 Oct 2;13:e19897. doi: 10.7717/peerj.19897 (PMC12497401; doi:10.7717/peerj.19897)
Supplement: Supplemental Information 2 — Measurements (in mm) to compare proportions of the ulna of Thalassocnus spp. (modified from Amson et al., 2015a:table 10). [file peerj-13-19897-s002.docx]

| **TABLE S2.** Measurements (in mm) to compare proportions of the ulna of *Thalassocnus* spp. (modified from Amson et al., 2015a:table 10). | | | | | |
| --- | --- | --- | --- | --- | --- |
| **Taxon** | **Specimen no.** | **Depth at midshaft (D)** | **Total length (L)** | **D/L** | **Source** |
| *T. natans* | MNHN.F.SAS734 | 28.9 | 333 | 0.09 | Amson et al., 2015a |
|  | MPC 704-A | 26 | 301 | 0.09 | This work |
| *T. littoralis* | MNHN.F.SAS56 | 21.4 | 297 | 0.07 | Amson et al., 2015a |
|  | MNHN.F.SAS620 | 27.4 | 318 | 0.09 | Amson et al., 2015a |
| *T. carolomartini* | MUSM 1995 | 32.6 | 353.6 | 0.09 | Amson et al., 2015a |
| *T. yaucensis* | MUSM 37 | 36.8 | 300.5 | 0.12 | Amson et al., 2015a |
