# Supplementary material for: Aquatic sloths (Thalassocnus) from the Miocene of Chile and the evolution of marine mammal herbivory in the Pacific Ocean
Source: PeerJ. 2025 Oct 2;13:e19897. doi: 10.7717/peerj.19897 (PMC12497401; doi:10.7717/peerj.19897)
Supplement: Supplemental Information 3 — Measurements (in mm) to compare proportions of the femur of Thalassocnus spp. (modified from Amson et al., 2015b:table 4). [file peerj-13-19897-s003.docx]

| **TABLE S3.** Measurements (in mm) to compare proportions of the femur of *Thalassocnus* spp. (modified from Amson et al., 2015b:table 4). | | | | | |
| --- | --- | --- | --- | --- | --- |
| **Taxon** | **Specimen no.** | **Total length (L)** | **Width at midshaft (W)** | **L/W** | **Source** |
| *T. natans* | MNHN.F.SAS734 | 312.8 | 57.8 | 5.41 | Amson et al., 2015b |
|  | MUSM 1916 | 339.5 | 55.7 | 6.10 | Amson et al., 2015b |
|  | MPC 704-A | 313 | 47 | 6.66 | This work |
|  | MPC 644 | 292 | 46 | 6.35 | This work |
| *T. littoralis* | MNHN.F.SAS40 | 305.1 | 49.1 | 6.21 | Amson et al., 2015b |
|  | MNHN.F.SAS41 | 283.7 | 54.5 | 5.21 | Amson et al., 2015b |
|  | MNHN.F.SAS42 | 264.6 | 38.7 | 6.84 | Amson et al., 2015b |
|  | MNHN.F.SAS53 | 288.4 | 41.5 | 6.95 | Amson et al., 2015b |
|  | MNHN.F.SAS158 | 273 | 50 | 5.46 | Amson et al., 2015b |
|  | MNHN.F.SAS1611 | 317.1 | 50.8 | 6.24 | Amson et al., 2015b |
|  | MNHN.F.SAS1621 | 290.8 | 46.5 | 6.25 | Amson et al., 2015b |
|  | MUSM 223 | 310 | 51.8 | 5.98 | Amson et al., 2015b |
| *T. carolomartini* | MUSM 1995 | 323.5 | 59.4 | 5.45 | Amson et al., 2015b |
| *T. yaucensis* | MUSM 37 | 401 | 73.2 | 5.48 | Amson et al., 2015b |
