# Supplementary material for: Aquatic sloths (Thalassocnus) from the Miocene of Chile and the evolution of marine mammal herbivory in the Pacific Ocean
Source: PeerJ. 2025 Oct 2;13:e19897. doi: 10.7717/peerj.19897 (PMC12497401; doi:10.7717/peerj.19897)
Supplement: Supplemental Information 4 — Measurements (in mm) to compare proportions of the femur and skull of Thalassocnus spp. (modified from Amson et al., 2015b:table 8). [file peerj-13-19897-s004.docx]

| **TABLE S4.** Measurements (in mm) to compare proportions of the femur and skull of *Thalassocnus* spp. (modified from Amson et al., 2015b:table 8). | | | | | |
| --- | --- | --- | --- | --- | --- |
| **Taxon** | **Specimen no.** | **Total length (Lf)** | **Toothrow length M1-M4 (Ls)** | **Lf/Ls** | **Source** |
| *T. natans* | MNHN.F.SAS734 | 312.8 | 50.7 | 6.17 | Amson et al., 2015b |
|  | MPC 704-A | 313 | 54 | 5.80 | This work |
| *T. littoralis* | MUSM 223 | 310 | 53.7 | 5.77 | Amson et al., 2015b |
| *T. carolomartini* | MNHN.F.SAS203 | 257.8 | 58.5 | 4.41 | Amson et al., 2015b |
| *T. yaucensis* | MUSM 37 | 276 | 60.7 | 4.55 | Amson et al., 2015b |
