# Supplementary material for: Aquatic sloths (Thalassocnus) from the Miocene of Chile and the evolution of marine mammal herbivory in the Pacific Ocean
Source: PeerJ. 2025 Oct 2;13:e19897. doi: 10.7717/peerj.19897 (PMC12497401; doi:10.7717/peerj.19897)
Supplement: Supplemental Information 5 — Measurements (in mm) to compare proportions of the femur and tibia of Thalassocnus spp. (modified from Amson et al., 2015b:table 13). [file peerj-13-19897-s005.docx]

| **TABLE S5.** Measurements (in mm) to compare proportions of the femur and tibia of *Thalassocnus* spp. (modified from Amson et al., 2015b:table 13). | | | | | |
| --- | --- | --- | --- | --- | --- |
| **Taxon** | **Specimen no.** | **Total length of femur (Lf)** | **Total length of tibia (Lt)** | **Lt/Lf** | **Source** |
| *T. natans* | MNHN.F.SAS734 | 312.8 | 274 | 0.88 | Amson et al., 2015b |
|  | MPC 704-A | 313 | 267 | 0.85 | This work |
| *T. littoralis* | MNHN.F.SAS53 | 288.4 | 271 | 0.94 | Amson et al., 2015b |
|  | MUSM 223 | 310 | 281 | 0.91 | Amson et al., 2015b |
| *T. carolomartini* | MUSM 1995 | 323.5 | 276 | 0.85 | Amson et al., 2015b |
