# Supplementary figures and images for: Aquatic sloths (Thalassocnus) from the Miocene of Chile and the evolution of marine mammal herbivory in the Pacific Ocean
Source: PeerJ. 2025 Oct 2;13:e19897. doi: 10.7717/peerj.19897 (PMC12497401; doi:10.7717/peerj.19897)

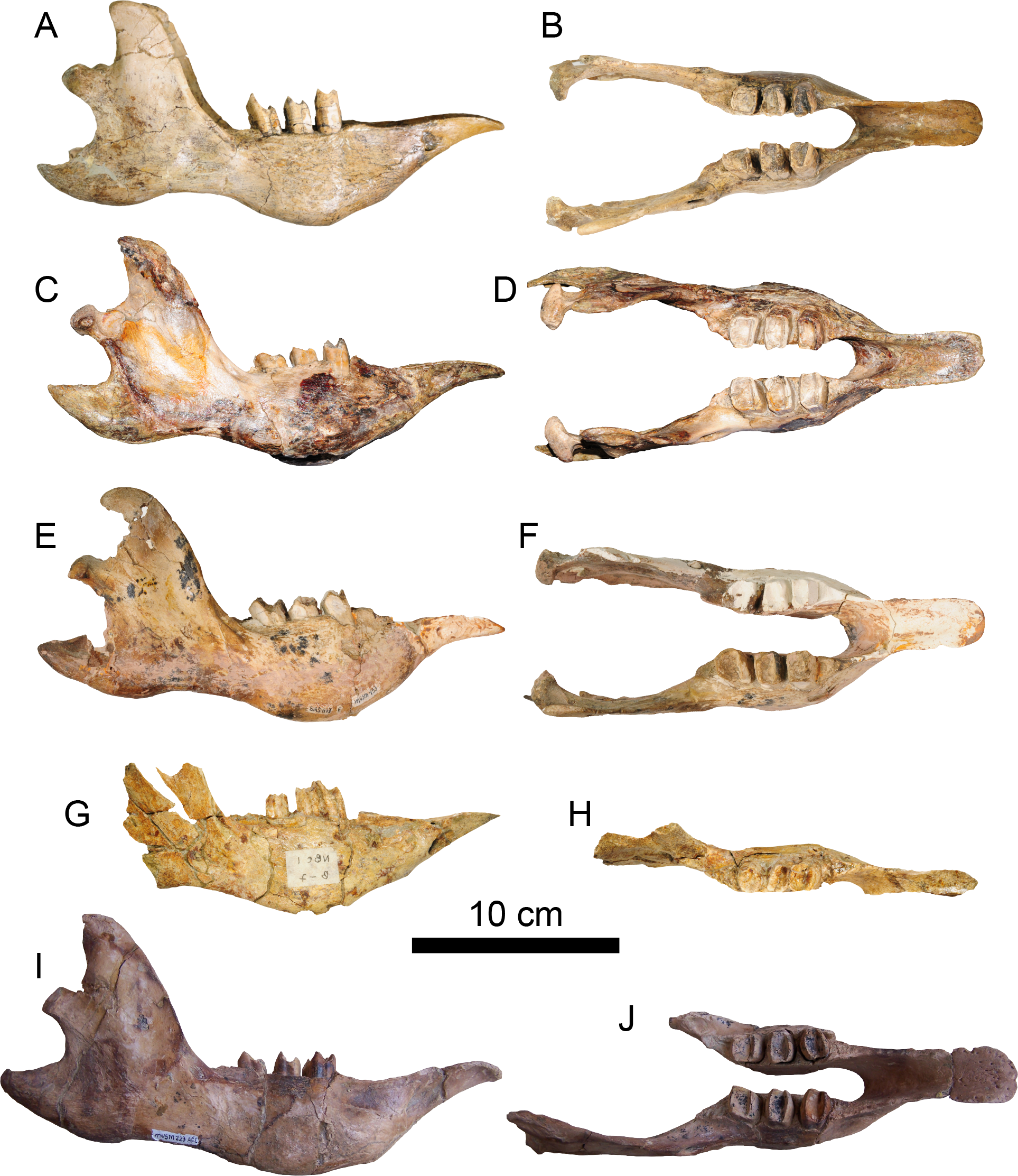

Supplement: Supplemental Information 7 — Thalassocnus antiquus (MUSM 228) in right lateral (A) and dorsal (B), views; T. natans (MNHN.F.SAS 734) in right lateral (C) and dorsal (D), views; T. natans (MUSM 433) in right lateral (E) and dorsal (F), views; left mandibular ramus of T. natans (MPC 705) in lateral (G) and dorsal (H), views; T. littoralis (MUSM 223) in right lateral (I) and dorsal (J), views. MPC 705 reversed for ease of comparison. [file peerj-13-19897-s007.png]

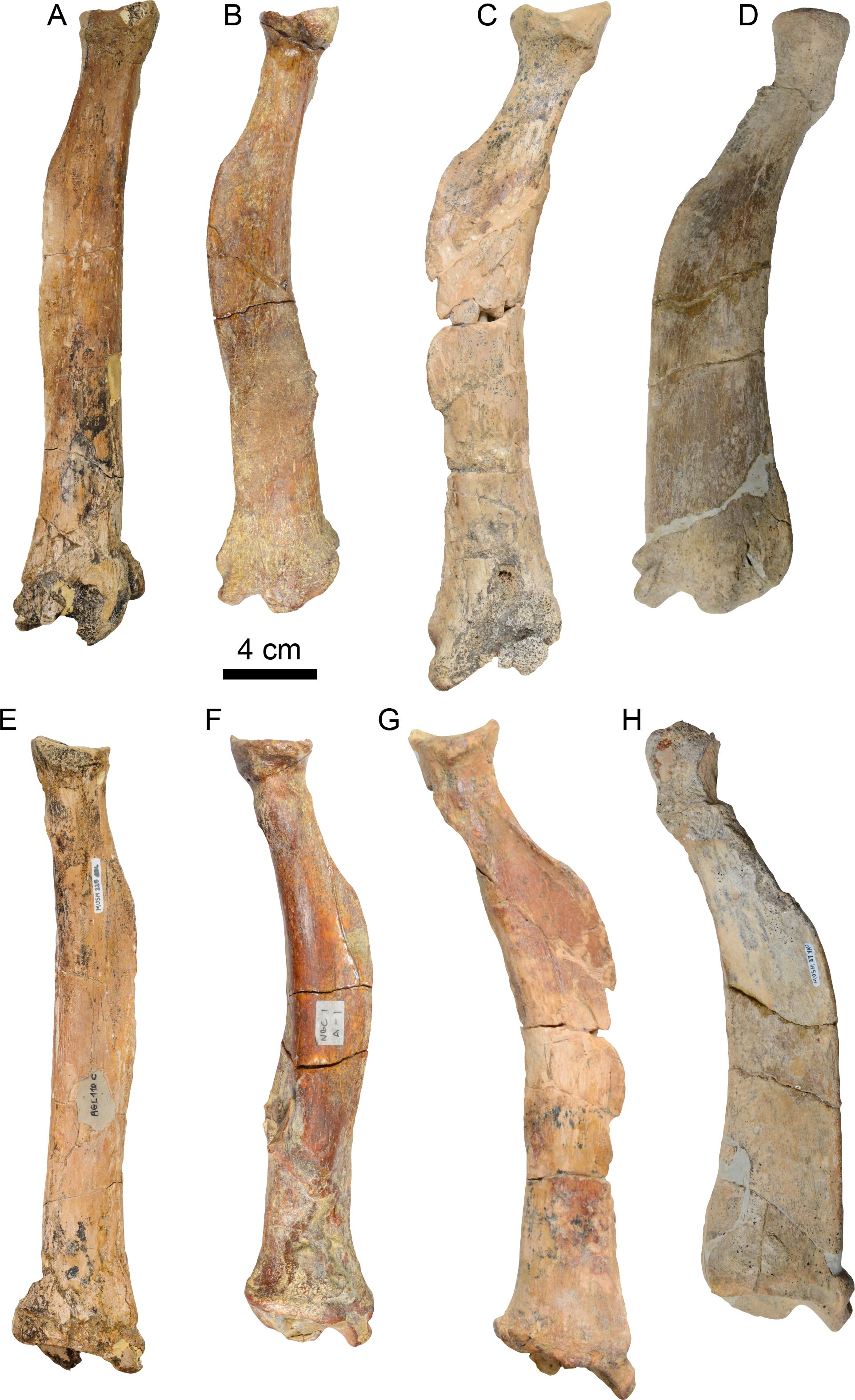

Supplement: Supplemental Information 8 — Left radius (MUSM 228) of Thalassocnus antiquus in lateral (A) and medial (E), views; left radius (MPC 704-A) of T. natans in lateral (B) and medial (F), views; left radius (MUSM 223) of T. littoralis in lateral (C) and medial (G), views; left radius (MUSM 37) of T. yaucensis in lateral (D) and medial H), views. [file peerj-13-19897-s008.png]

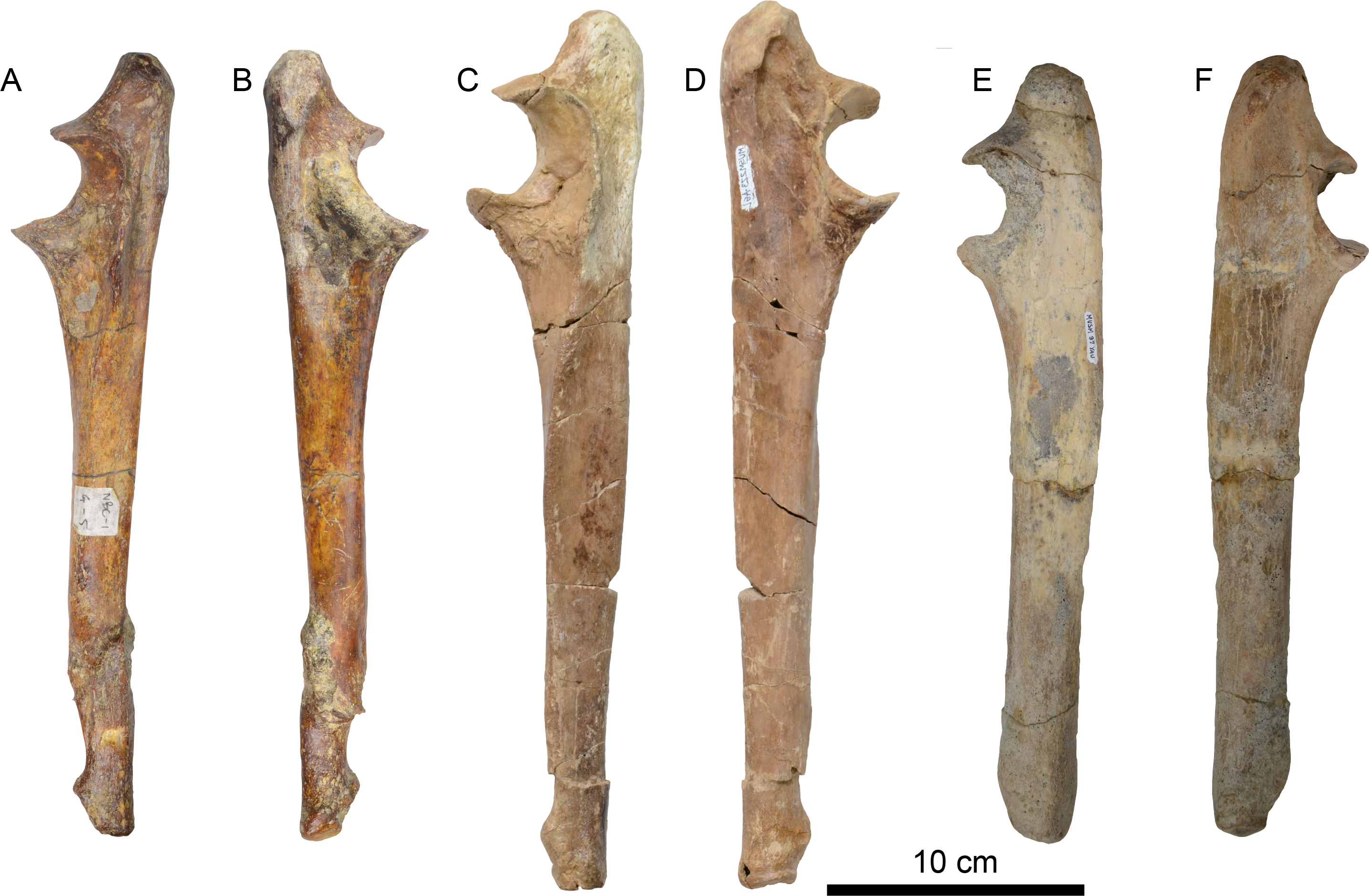

Supplement: Supplemental Information 9 — Left ulna (MPC 704-A) of Thalassocnus natans in lateral (A) and medial (B), views; right ulna (MUSM 223) of T. littoralis in lateral (C) and medial (D), views; left ulna (MUSM 37) of T. yaucensis in lateral (E) and medial (F), views. MUSM 223 reversed for ease of comparison. [file peerj-13-19897-s009.png]

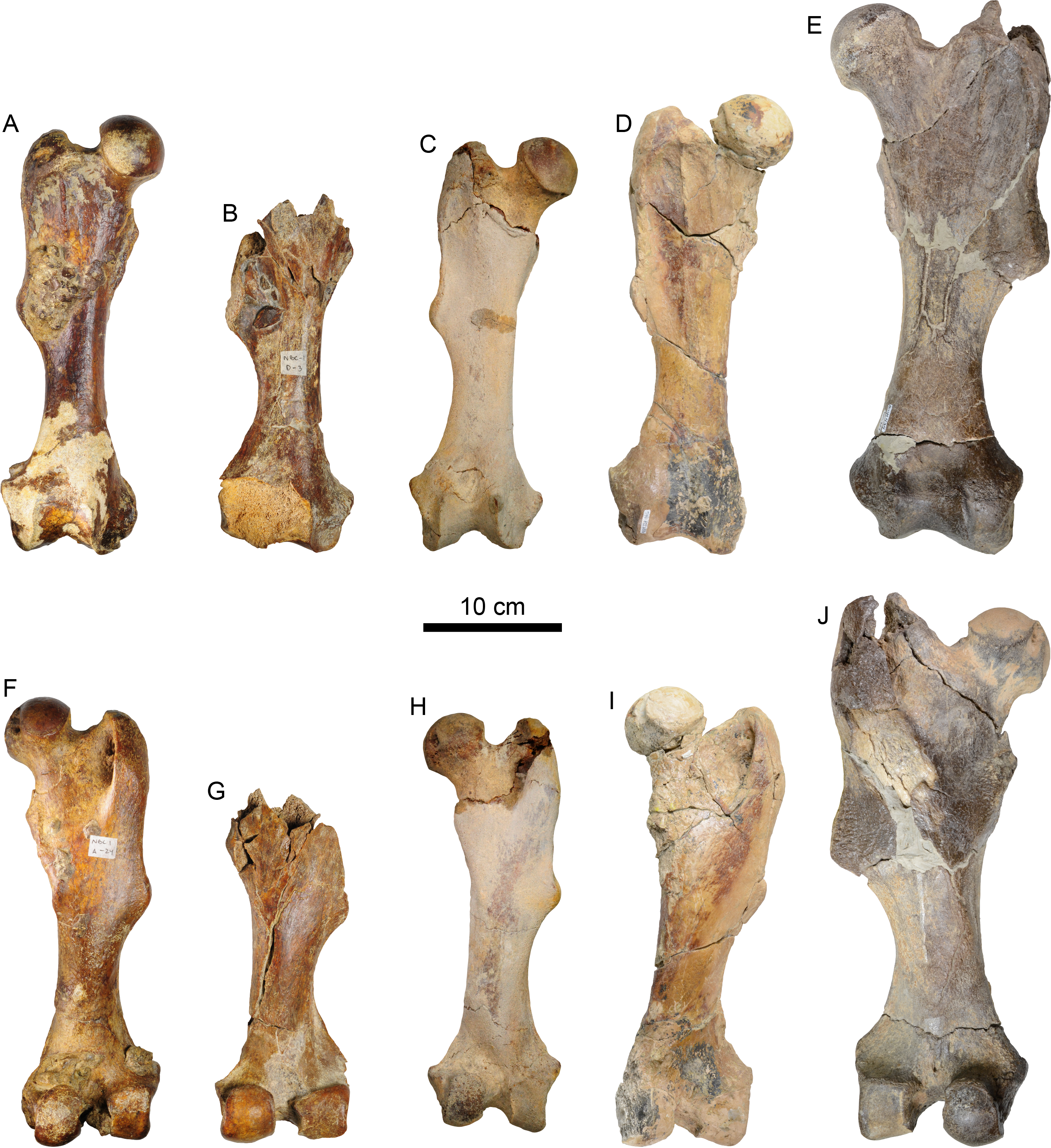

Supplement: Supplemental Information 10 — Right femur (MPC 704-A) of Thalassocnus natans in anterior (A) and posterior (F), views; right femur (MPC 705-A) of T. natans in anterior (B) and posterior (G), views; right femur (MPC 644) of T. natans in anterior (C) and posterior (H), views; right femur (MUSM 223) of T. littoralis in anterior (D) and posterior (I), views; left femur (MUSM 434) of T. yaucensis in anterior (E) and posterior (J), views. MUSM 434 reversed for ease of comparison. [file peerj-13-19897-s010.png]
